# Examining associations of folic acid supplements administered to mothers during pre-conceptional and prenatal periods with autism spectrum disorders in their offspring: insights from a multi-center study in China

**DOI:** 10.3389/fpubh.2024.1321046

**Published:** 2024-01-17

**Authors:** Yan Jiang, Cuihua Guo, Min Kuang, Lizi Lin, Guifeng Xu, Ning Pan, Xuchu Weng, Jin Jing, Lei Shi, Quanying Yi, Xin Wang

**Affiliations:** ^1^Department of Children Health Care, Dongguan Children's Hospital, Dongguan, Guangdong, China; ^2^Department of Occupational and Environmental Health, Guangdong Provincial Engineering Technology Research Center of Environmental Pollution and Health Risk Assessment, School of Public Health, Sun Yat-sen University, Guangzhou, Guangdong, China; ^3^The First Affiliated Hospital, University of Science and Technology of China, Hefei, Anhui, China; ^4^Key Laboratory of Brain, Cognition and Education Sciences, Ministry of Education; Institute for Brain Research and Rehabilitation, and Guangdong Key Laboratory of Mental Health and Cognitive Science, South China Normal University, Guangzhou, Guangdong, China; ^5^Department of Maternal and Child Health, Research Center of Children and Adolescent Psychological and Behavioral Development, School of Public Health, Sun Yat-sen University, Guangzhou, Guangdong, China; ^6^JNU-HKUST Joint Laboratory for Neuroscience and Innovative Drug Research, College of Pharmacy, Jinan University, Guangzhou, Guangdong, China

**Keywords:** autism spectrum disorder, folic acid, pre-conceptional period, prenatal period, environmental factor

## Abstract

**Objective:**

To investigate the relationship between maternal folic acid (FA) supplementation during the pre-conceptional and prenatal periods and the subsequent risk of autism spectrum disorder (ASD) in offspring.

**Methods:**

A total of 6,049 toddlers aged 16–30 months were recruited from August 2016 to March 2017 for this cross-sectional study conducted in China. The parents of the enrolled toddlers provided information on maternal supplemental FA, socio-demographic information, and related covariates. Standard diagnostic procedures were implemented to identify toddlers with ASD.

**Results:**

Among the 6,049 children included in the study, consisting of 3,364 boys with an average age of 22.7 ± 4.1 months, a total of 71 children (1.2%) were diagnosed with ASD. Mothers who did not consume FA supplements during the prenatal period were found to have a significantly increased risk of having offspring with ASD, in comparison to those who were exposed to FA supplements (odds ratio [*OR*] = 2.47). However, we did not find a similar association during the pre-conceptional period. Compared to mothers who consistently used FA supplements from pre-conception to the prenatal period, those who never used FA supplements were statistically significantly associated with a higher risk of ASD in their offspring (*OR* = 2.88).

**Conclusion:**

This study indicated that providing continuous maternal FA supplementation during the pre-conceptional and prenatal periods may decrease the risk of ASD in offspring. The prenatal period is considered to be the most crucial time for intervention.

## 1 Introduction

Autism spectrum disorder (ASD) is a neurodevelopmental disorder characterized by social communication challenges and restricted, repetitive patterns of behaviors, interests, or activities (1). The multifactorial theory of ASD risk has been widely accepted (2), involving the jointly mediated role of environmental factors and genetic predispositions (3, 4). Environmental factors may trigger predisposing hereditary high-risk gene modifications during fetal development through epigenetic mechanisms (3, 5). Emerging evidence suggests that folate, as a modifiable risk factor, may play an important role in the etiology of ASD (6, 7).

Folate, one water-soluble substance belonging to the vitamin B family, participates in various important reactions, such as DNA methylation and replication, during multiple physiological processes (6). Cell folate, as one of the methyl donors, exerts its influence on the developing brain through the synthesis of DNA, neurotransmitters, and myelination (6). Mechanism-related studies have revealed that folate might provide preventive effects for ASD and other neurological diseases, such as neural tube defects (NTDs) (6). Epidemiological studies have already investigated the potential causal association between folate intake and the onset of ASD in offspring, but the results have been with conflicting (8–19) (See Supplementary Table 1). One recent meta-analysis study, which included a total of 10 studies with 23 sub-studies (9,795 ASD cases), found that supplementing with FA during early pregnancy was associated with a lower risk of ASD in offspring (*OR =* 0.57, 95% *CI*: 0.41–0.78), while consuming a daily amount of at least 400 μg from dietary sources and supplements was also associated with a reduced risk of offspring ASD (*OR* = 0.55, 95% *CI*: 0.36–0.83) (4). However, the results showed high heterogeneity, and meta-regression analyses indicated that countries and supplementary timings may be significant source of heterogeneity. Most of the aforementioned studies were conducted in Western countries, while few studies were performed in China, yielding mixed results (16, 19). A case–control study from China showed that children born to mothers who did not use FA supplementation had an increased risk (1.905, 95%*CI*, 1.238–2.933) compared to those who used 400 μg FA daily for a duration of 24 weeks [i.e., starting 12 weeks before the last menstrual period (LMP) and continuing for 12 weeks after the LMP] (19). However, Li et al. (16) found no association between FA supplementation during pregnancy preparation, pregnancy or lactation, and the risk of offspring ASD. These findings indicate that the timing of the FA supplement may affect the associations between FA and the risk of ASD in offspring, which is still unclear.

Given this background, we conducted a nationwide cross-sectional study to distinguishing the timing of FA supplementation during the pre-conceptional and prenatal period. Our aim is to investigate the associations between different timing of FA supplementation and the odds of ASD in Chinese toddlers.

## 2 Methods

### 2.1 Study population and overall design

We used a cross-sectional study design. From August 2016 to March 2017, we conducted a national validation study of the Chinese version of the Modified Checklist for Autism in Toddlers, Revised with Follow-Up (M-CHAT-R/F) in China. We implemented a convenient cluster-sampling strategy and gathered the sample from seven cities in six provinces across five geographical regions of China. The cities included Beijing City (Northern region), Chongqing City, and Guiyang City in Guizhou Province (Western region), Guangzhou City, and Foshan City in Guangdong Province (Southern region), Wuhan City in Hubei Province (Central region), and Hangzhou City in Zhejiang Province (Eastern region). In each sampling site, we recruited study samples from a variety of sources, including hospital-based, community-based, and school-based populations. Finally, we recruited a total of 7,928 caregivers who agreed to participate in the validation study, along with their children aged 16–30 months. The participants were selected from seven tertiary hospitals, 21 communities, and seven kindergartens. At the same time as the validation study, an additional survey was conducted for all 7,928 caregivers of the toddlers, of which 6,049 agreed to complete questionnaires (The derivation of the study sample was shown in Supplementary Figure 1). The demographic characteristics of participants who completed or did not complete the questionnaires were compared in Supplementary Table 2. All the 6,049 mothers and their children from our validation study were included in this study. The further details of the study could be referred from a published article (20).

We followed the reporting guideline for cross-sectional studies according to the Strengthening the Reporting of Observational Studies in Epidemiology (STROBE) (21). We have obtained the ethical approval from the Ethical Review Committee for Biomedical Research at Sun Yat-sen University. All participants provided written informed consent, which informed them of the purpose of the study.

### 2.2 Procedure of ASD screening and diagnosis

We adopted a two-stage process to confirm the ASD diagnosis of the included population. In the first stage, caregivers completed the M-CHAT-R/F during the well-child visit. The M-CHAT-R/F consists of 20 questions regarding the presence or absence of symptoms related to autism. A total score ranging from 0 to 2 indicates a low risk. Children who failed three out of the 20 items in the M-CHAT-R were considered at risk for autism (22). In the second stage, children who screened positive on M-CHAT-R/F or whose caregiver or physician subsequently expressed concern were referred by their physician for a diagnostic evaluation. Diagnostic evaluations were conducted, which included face-to-face assessments using the Childhood Autism Rating Scale (23), as well as 30 min of parent interviews and interactions with toddlers. These evaluations were carried out by licensed child psychiatrists and trained psychologists. The final diagnosis was made based on a 30-min interaction and the criteria outlined in the Statistical Manual of Mental Disorders, Fifth Edition (1).

### 2.3 Assessment of FA supplementation

Folic acid supplement consumption during the pre-conceptional and prenatal periods was reported by caregivers. We defined FA supplementation during the pre-conceptional period as taking FA supplements for at least 3 months before becoming aware of pregnancy. The answer was a binary variable encoded as 0 for “no” and 1 for “yes.” FA supplementation during the prenatal period was defined and coded in a similar way.

We further categorized the mothers into three groups: continuously FA users, who took FA supplements during both the pre-conceptional and prenatal periods; ever FA users, who took FA supplements during either the pre-conceptional or prenatal period; and FA nonusers, who never took FA supplements. The details of three groups were shown in Supplementary Figure 2.

### 2.4 Statistical analysis

T-tests were used to compare the characteristics of continuous variables, while chi-square analysis was performed to compare the characteristics of categorical variables between children with and without ASD. We estimated the odds ratios (ORs) and 95% confidence intervals (CI) of ASD based on the maternal FA supplementation status using multivariable logistic regression. We first adjusted for child age, child sex, whether there was only one child, maternal age, maternal education level, annual household income, research area, and ethnic background. In model 2, we further adjusted for gestational age, complications during pregnancy, depressive symptoms during pregnancy, secondhand smoke exposure during pregnancy, and pre-pregnancy overweight/obesity. All covariates were selected based on their association with ASD or the use of FA supplements (24, 25).

We conducted several sensitivity analyses: (1) we re-ran the models using Firth’s bias-reduced logistic regression (26). Firth’s bias reduction method corrects bias in small-sample logistic regression by adding a penalty term to the maximum likelihood estimation. In situations with limited events or rare outcomes, traditional methods yield imprecise and biased results. Firth’s approach introduces a profiled Jeffreys prior as a penalty, stabilizing estimation and mitigating small-sample bias. This method improves parameter accuracy by adjusting the likelihood function, making it particularly valuable when dealing with sparse data or infrequent events (27). (2) We repeated the primary analysis separately for male offspring, full-term offspring, offspring of mothers without pregnancy complications, and offspring of mothers without overweight/obesity prior to pregnancy. We did not conduct stratified analyses because the number of ASD cases was small.

Statistical analyses were conducted using the statistical software R, version 3.6.1. All tests conducted were bilateral tests, and a *p* value of less than 0.05 was considered statistically significant.

## 3 Results

### 3.1 Demographic characteristics and maternal risk factors during pregnancy

The mean age of the children at the time of screening was 22.7 months (range 16–30 moths), and there were slightly more boys than girls (55.6 vs. 44.4%), as shown in Table 1. Additionally, 70.7% of the children were the only child in their family. Most of them were full-term babies (91.6%) and of Han nationality (93.8%). The mean age of the mothers was 30.4 ± 4.1 years. Most of the mothers were well-educated, with 67.5% having at least an undergraduate university degree. Few mothers reported complications during pregnancy (14.2%), obesity/overweight before pregnancy (7.8%), or second-hand smoke exposure during pregnancy (13.9%). About three-quarters of mothers did not feel depressed during pregnancy. We identified 71 cases of ASD in our survey study sample, which accounted for 1.2% of the total. Male gender, lower levels of maternal education, depressive symptoms during pregnancy, and exposure to second-hand smoke were more frequently observed in cases of ASD in this study (Table 1).

**Table 1:** Demographic characteristics of the 6,049 participants in this study.

Characteristic		*N* (%)/Mean (SD)			*p* value
		Children without ASD (*N* = 5,978)	Children with ASD (*N* = 71)	Overall (*N* = 6,049)	
Child age (in months)		22.7 ± 4.1	24.3 ± 3.6	22.7 ± 4.1	**< 0.01**
Gender^a^	Boy	3,302 (55.2)	62 (87.3)	3,364 (55.6)	**< 0.01**
	Girl	2,676 (44.8)	9 (12.7)	2,685 (44.4)	
Only child	Yes	4,229 (70.7)	48 (67.6)	4,277 (70.7)	0.56
	No	1749 (29.3)	23 (32.4)	1772 (29.3)	
Preterm birth	No	5,475 (91.6)	67 (94.4)	5,542 (91.6)	0.40
	Yes	503 (8.4)	4 (5.6)	507 (8.4)	
Ethnic background	Han	5,605 (93.8)	69 (97.2)	5,674 (93.8)	0.23
	Minority	373 (6.2)	2 (2.8)	375 (6.2)	
Yearly household income ^b^	≤￥100, 000	2,122 (35.5)	31 (43.7)	2,153 (35.6)	0.35
	￥100, 000–300, 000	2,293 (38.4)	21 (29.6)	2,314 (38.3)	
	≥ ￥300, 000	457 (7.6)	7 (9.9)	464 (7.7)	
	Not reported	1,106 (18.5)	12 (16.9)	1,118 (18.5)	
Study area	Beijing	1,307 (21.9)	1 (1.4)	1,308 (21.6)	**< 0.01**
	Guangdong	2,541 (42.5)	55 (77.5)	2,596 (42.9)	
	Zhejiang	921 (15.4)	4 (5.6)	925 (15.3)	
	Chongqing	1,091 (18.3)	6 (8.5)	1,097 (18.1)	
	Hubei	118 (2.0)	5 (7.0)	123 (2.0)	
Maternal age (in years)	≤24 years	1,031 (17.2)	14 (19.7)	1,045 (17.3)	0.94
	25–29 years	3,151 (52.7)	37 (52.1)	3,188 (52.7)	
	30–34 years	1,391 (23.3)	16 (22.5)	1,407 (23.3)	
	≥35 years	405 (6.8)	4 (5.6)	409 (6.8)	
Maternal education	Primary school and below	661 (11.1)	16 (22.5)	677 (11.2)	**< 0.01**
	Middle school	1,269 (21.2)	20 (28.2)	1,289 (21.3)	
	College degree	3,737 (62.5)	31 (43.7)	3,768 (62.3)	
	Advanced degree	311 (5.2)	4 (5.6)	315 (5.2)	
Complication during pregnancy	No	5,129 (85.8)	60 (84.5)	5,189 (85.8)	0.76
	Yes	849 (14.2)	11 (15.5)	860 (14.2)	
Depressive symptom during pregnancy	No	4,519 (75.6)	44 (62.0)	4,563 (75.4)	**< 0.01**
	Yes	1,459 (24.4)	27 (38.0)	1,486 (24.6)	
Overweight/obesity during pre-pregnancy	No	5,513 (92.2)	64 (90.1)	5,577 (92.2)	0.52
	Yes	465 (7.8)	7 (9.9)	472 (7.8)	
Second-hand smoke during pregnancy	No	5,153 (86.2)	53 (74.6)	5,206 (86.1)	
	Yes	825 (13.8)	18 (25.4)	843 (13.9)	**< 0.01**

ASD, Autism spectrum disorder. ^a^2016 census data on sex ratio of children aged 0–4 years: 116.23:100. ^b^2016 census data: per-capita urban disposable income is ￥31,194.8; per-capita rural disposable income is ￥11,421.7. Source: China Statistical Yearbook 2016.

### 3.2 Prevalence of ASD associated with FA supplements

**Figure 1:**
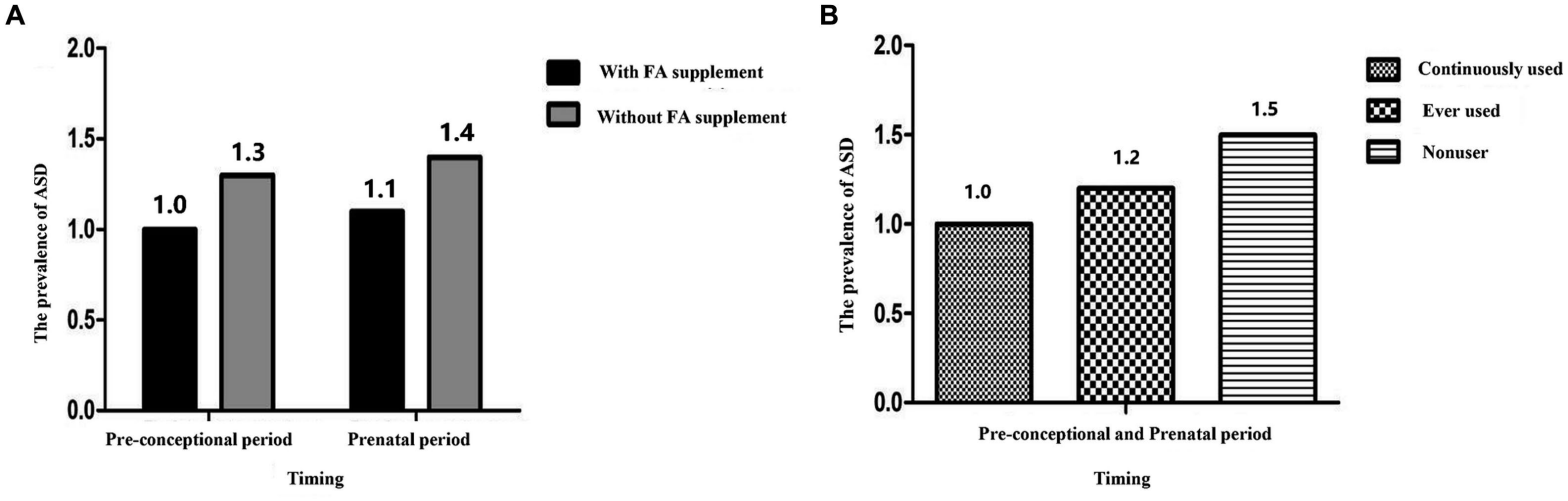
The prevalence of ASD with different timing of FA supplement. **(A)** The prevalence of ASD with FA supplement during the pre-conceptional and prenatal period; **(B)** the prevalence of ASD with continuously used, ever used, nonuser. FA, Folic acid; ASD, Autism spectrum disorder.

Figure 1 showed the prevalence of ASD with different timing of FA supplementation. The prevalence of ASD would be higher if mothers did not take FA supplements during the pre-conceptional and prenatal periods (For specific values, see Supplementary Table 3). From the pre-conceptional to prenatal period, the prevalence of ASD in offspring was lowest in mothers who continuously used FA supplements, followed by those who had ever used them. For mothers who did not take FA supplements, the prevalence of their children was highest.

### 3.3 FA supplements and the risk of ASD

We assessed the potential association between FA supplementation and the development of ASD in various time. During the pre-conceptional period, there appeared to be no detectable association between FA supplementation and the risk of offspring ASD, as indicated by both the crude model and the adjusted model (Table 2). However, during the prenatal period, the adjusted *odds ratio* (*OR*) was 2.47 (95% *CI*, 1.37–4.31) and 2.54 (95% *CI*, 1.41–4.44) after adjusting for covariates in model 1 and model 2, respectively (Table 2). We further grouped maternal FA supplements into three categories: continuously used, ever used, and nonuser. Compared to the continuously used group, the nonuser group had a higher risk of ASD in the offspring (adjusted *OR* was estimated at 2.88 [95% *CI*, 1.46–5.55] and 2.93 [95% *CI*, 1.48–5.67] in model 1 and model 2, respectively). Interestingly, the risk of offspring ASD was not significant when mothers took FA supplements either during the pre-conceptional or prenatal period. Even after adjustment, the adjusted *OR* of ASD in the ever-used group compared with the continuously used group was estimated at 1.24 (95% *CI*, 0.70–2.18) and 1.22 (95% *CI*, 0.69–2.16) in model 1 and model 2.

**Table 2:** Associations of maternal FA supplementation and ASD among toddlers (16–30 months).

Timing of FA supplement	Crude model^a^		Adjusted model 1^b^		Adjusted model 2^c^	
	OR (95%CI)	*p* value	OR (95%CI)	*p* value	OR (95%CI)	*p* value
Pre-conceptional period						
Yes	1[Reference]		1[Reference]		1[Reference]	
No	1.30 (0.81, 2.09)	0.27	1.49 (0.92, 2.43)	0.11	1.47 (0.90, 2.41)	0.12
Prenatal period						
Yes	1[Reference]		1[Reference]		1[Reference]	
No	1.26 (0.75, 2.04)	0.37	**2.47 (1.37, 4.31)**	**< 0.001**	**2.54 (1.41, 4.44)**	**< 0.001**
Pre-conceptional and prenatal period						
Continuously used	1[Reference]		1[Reference]		1[Reference]	
Ever used	1.14 (0.65, 1.96)	0.65	1.24 (0.70, 2.18)	0.46	1.22 (0.69, 2.16)	0.49
Nonuser	1.45 (0.80, 2.58)	0.21	**2.88 (1.46, 5.55)**	**< 0.001**	**2.93 (1.48, 5.67)**	**< 0.001**

FA, Folic acid; OR, Odds ratio; and CI, Confidence intervals. ^a^Crude model: adjusted for nothing. ^b^ Adjusted model 1: adjusted for child age, gender, only child, maternal age, maternal education level, annual household income, research area, and ethnic background. ^c^Adjusted model 2: further adjusted for preterm birth, overweight/obesity before pregnancy, pregnant complications, depressive symptoms during pregnancy, and secondhand smoke during pregnancy.

### 3.4 Sensitivity analyses

Considering the small number of ASD cases in our study, we re-run the models using Firth’s bias reduction method. The results were consistent with the primary analysis (see Supplementary Table 4). We also conducted a separate analysis focusing on male offspring, full-term offspring, offspring of mothers without pregnancy complications, and offspring of mothers without overweight/obesity before pregnancy (see Supplementary Tables 5–8). The results were consistent with previous findings, suggesting that maternal exposure to FA supplements during pregnancy was significantly associated with a reduced risk of ASD.

## 4 Discussion

In the nationwide cross-sectional study conducted in China, we found that mothers who did not take FA supplements during the prenatal period had a higher risk of having offspring with ASD compared to those who did take FA supplements. However, we did not observe any significant association during the pre-conceptional period. However, mothers without FA supplements during both pre-conceptional and prenatal periods showed higher risks of having offspring with ASD compared to those who took continuous FA supplements.

This study indicated that maternal FA supplementation during the pre-conceptional and prenatal periods might have a protective effect on ASD in offspring. Additionally, the prenatal period might be the more critical time window for supplementation. Our results were consistent with several previous studies that found a protective role of prenatal FA supplementation. These studies include the Stockholm Youth Cohort Study (14) and the Norwegian Mother and Child Cohort Study, specifically the Autism Birth Cohort (ABC) sub-study (10). However, a case–control study from China with 374 children diagnosed with ASD and 354 typically developing children found no association during either the pre-conceptional or prenatal period (16). On the other hand, a case–control cohort study of 45,300 Israeli children found that both pre-conceptional and prenatal FA supplements reduce the risk of ASD in the offspring (15). In China, women who are planning a pregnancy are recommended to take FA starting from 3 months before conception until the end of the first trimester (28) in order to prevent NTDs. However, we found that only 46.5% of women continuously took FA supplements during the pre-conceptional and prenatal periods, and approximately one-third of women ever took FA supplements during either the pre-conceptional or prenatal periods. For women who have ever taken FA supplement, we did not find an increased risk of their offspring developing ASD compared to those who consistently took FA supplements. A potential explanation could be that the folate level of Chinese women was insufficient because food fortification with FA has not yet been implemented in China (29). For instance, the median serum folate concentrations in Chinese women, as reported by the Chinese Center for Disease Control and Prevention in 2015, were 8.02 ng/mL, which is significantly lower than the levels found in developed countries like the United States (14.9–16.8 ng/mL) (30) and New Zealand (21.4 ng/mL) (31). Therefore, taking FA supplements at any time during the pre-conceptional or prenatal period might help to achieve adequate serum folate concentrations (29).

In addition, although a previous meta-analysis (4) revealed that consuming FA supplements during periconception or early pregnancy could reduce the risk of ASD in offspring, emerging evidence shows that low plasma/serum folate concentration in the second and third trimesters of pregnancy is also likely associated with increased risks of physical health issues (e.g., low birthweight) (32) and cognitive development (e.g., language development) in offspring (33, 34). Folate plays an essential role in DNA methylation as a provider of methyl groups (6), which is considered crucial for the development of the central nervous system and is implicated in the formation of fundamental brain structures (15). There might be different windows of susceptibility to maternal changes in the folate-dependent 1-carbon pathway. The periods beyond periconception may play important roles in influencing epigenetic changes in the offspring (35). Therefore, our findings suggested that FA supplements should be taken during the pre-conception period for women who are planning to become pregnant. For women with unintended pregnancies, it is recommended to continue taking FA supplements during the prenatal period.

Our findings have important clinical implications for the relevant policy and practice of FA supplementation in China. In 2009, China’s Ministry of Health launched a major public health project to provide women with FA supplements from 3 months before pregnancy to the first trimester in order to prevent NTDs. After the implementation of the FA intervention project, the rate of pregnant women taking FA supplements increased significantly. However, the rate of women planning pregnancy who take FA supplements as recommended is still low, ranging from 10 to 35% (36). Social-economic status, unintended pregnancy, and awareness and belief about FA supplement were the main influencing factors for taking it as recommended (37). Our results highlighted that the critical window for protecting offspring from ASD is the prenatal period. Policy-led interventions, such as health education, are still urgently needed in China to encourage women to insist on taking folic acid supplements from pre-conception to the prenatal period. In addition, healthcare professionals should also suggest and encourage expectant parents to take FA supplements routinely when they are preparing for pregnancy.

This study had one important strength: it used standard procedures for ASD screening and diagnosis to ensure accurate outcome measurements. However, several limitations should be noted. First, the prevalence of ASD was found to be low in this large epidemiological survey. However, the prevalence in this study was similar to the national estimate in China (37). In addition, we have applied relevant statistical analyses, such as Firth’s bias-reduced regression, to address this problem. Second, the use of questionnaires might introduce potential recall bias, but this remains the most effective investigation method in large epidemiological studies. Thirdly, we did not collect information on the dosage of the FA supplement, and we did not measure maternal serum folate levels. However, doctors usually give advice with a fixed dose as a recommendation to women who regularly take FA supplements during the preconception and prenatal period. Further studies are needed to investigate the dose–response associations during the pre-conceptional and prenatal periods. Furthermore, it should be noted that this study employed a cross-sectional design, which limits the ability to establish causality. Moreover, it is important to acknowledge that other nutritional supplements during pregnancy that could confound and interact the results of the current study, such as iron intake (38), vitamin D deficiency (39), or multivitamin supplementation. Additionally, despite we have considered to fit mix-effect models to preclude the random effects, the small sample size of individuals with ASD prevented the models from converging effectively, thus hindering our ability to confirm the presence of unobserved heterogeneity. A larger sample size with more diagnosed ASD will be needed to confirm the stability of results in the future.

## 5 Conclusion

According to the results of the current investigation, regular maternal use of FA supplements during the pre-conceptional and pre-natal phases may lower the incidence of ASD in offspring. Additionally, it was determined that the most crucial window of time for this intervention occurred during the prenatal period. The importance of maternal prenatal FA supplementation in lowering the risk of ASD in offspring is highlighted by our research. In order to get Chinese women to take FA supplements, urgent policy-led interventions and health promotion initiatives are required.

## Data availability statement

The raw data supporting the conclusions of this article will be made available by the authors, without undue reservation.

## Ethics statement

The studies involving humans were approved by the Ethical Review Committee for Biomedical Re-search, Sun Yat-sen University (2016-No.013). The studies were conducted in accordance with the local legislation and institutional requirements. Written informed consent for participation in this study was provided by the participants’ legal guardians/next of kin.

## Author contributions

YJ: Conceptualization, Data curation, Formal analysis, Investigation, Methodology, Resources, Software, Validation, Writing – original draft, Writing – review & editing. CG: Data curation, Formal analysis, Investigation, Methodology, Resources, Software, Validation, Writing – original draft, Writing – review & editing. MK: Data curation, Investigation, Writing – review & editing. LL: Funding acquisition, Resources, Software, Validation, Writing – review & editing. GX: Writing – review & editing. NP: Resources, Software, Validation, Writing – review & editing. XWe: Writing – review & editing. JJ: Funding acquisition, Project administration, Writing – review & editing. LS: Funding acquisition, Project administration, Writing – review & editing. QY: Conceptualization, Funding acquisition, Project administration, Supervision, Writing – review & editing. XWa: Conceptualization, Funding acquisition, Project administration, Supervision, Writing – review & editing.
