# Supplementary material for: Examining associations of folic acid supplements administered to mothers during pre-conceptional and prenatal periods with autism spectrum disorders in their offspring: insights from a multi-center study in China
Source: Front Public Health. 2024 Jan 17;12:1321046. doi: 10.3389/fpubh.2024.1321046 (PMC10827999; doi:10.3389/fpubh.2024.1321046)
Supplement: Supplementary file 1 [file Data_Sheet_1.docx]

Supplementary Material

**Supplementary Figure 1** Derivation of the study sample

**Supplementary Figure 2** The Flowchart of 3 groups categorized with folic acid supplement during pre-conceptional and prenatal period

**Supplementary Table 1** Summaries of previous observational studies regarding the associations of folic acid supplements with ASD

**Supplementary Table 2** Demographic characteristics of 7928 study participants with or without the information of questionnaires

**Supplementary Table 3** Prevalence of ASD associated with maternal folic acid supplement among toddlers aged16-30 Months

**Supplementary Table 4** The Firth’s Bias-Reduced Logistic Regression of the associations regarding pre-conceptional and prenatal folic acid supplement with ASD among toddlers aged 16-30 months

**Supplementary Table 5** Sensitivity analyses of the associations regarding pre-conceptional and prenatal folic acid supplement with ASD among toddlers aged 16-30 months restricting in boys

**Supplementary Table 6** Sensitivity analyses of the associations regarding pre-conceptional and prenatal folic acid supplement with ASD among toddlers aged 16-30 months restricting in full-term infants

**Supplementary Table 7** Sensitivity analyses of the associations regarding pre-conceptional and prenatal folic acid supplement with ASD among toddlers aged 16-30 months with mothers of no complications during pregnancy

**Supplementary Table 8** Sensitivity analyses of the associations regarding pre-conceptional and prenatal folic acid supplement with ASD among toddlers aged 16-30 months with mothers of no Pre-pregnancy overweight/obesity

**
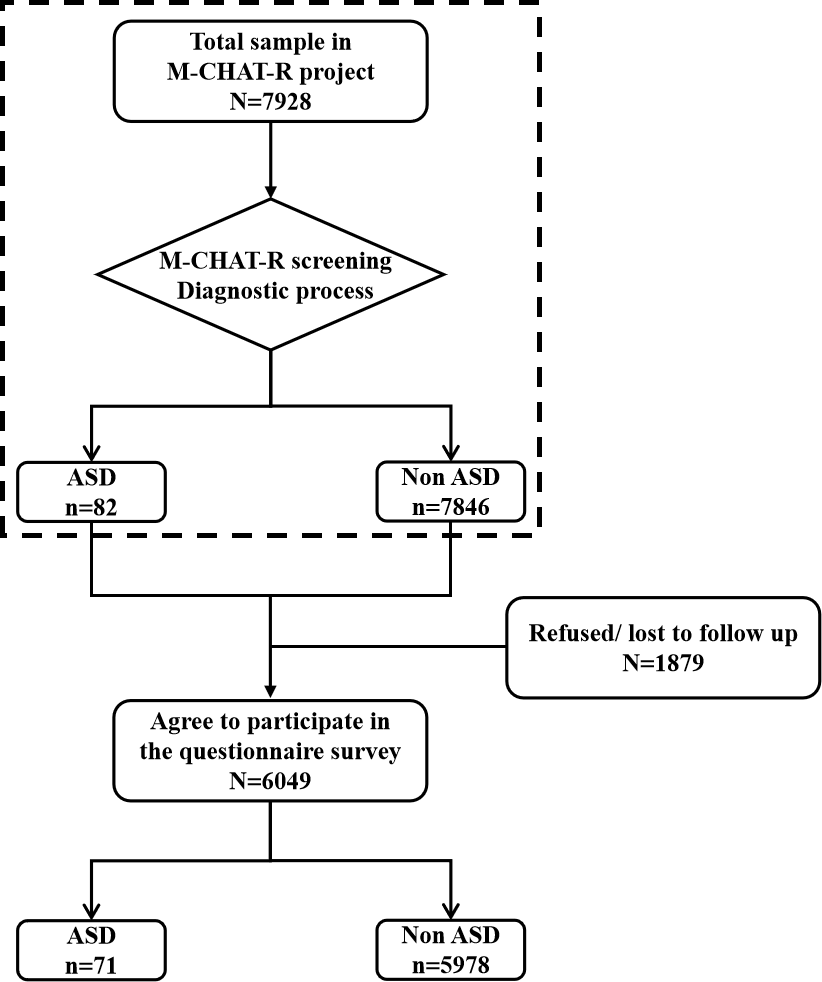
**

**Abbreviations**: M-CHAT-R, the Modified Checklist for Autism in Toddlers; ASD: autism spectrum disorder.

**Supplementary Figure 1.** Derivation of the study sample


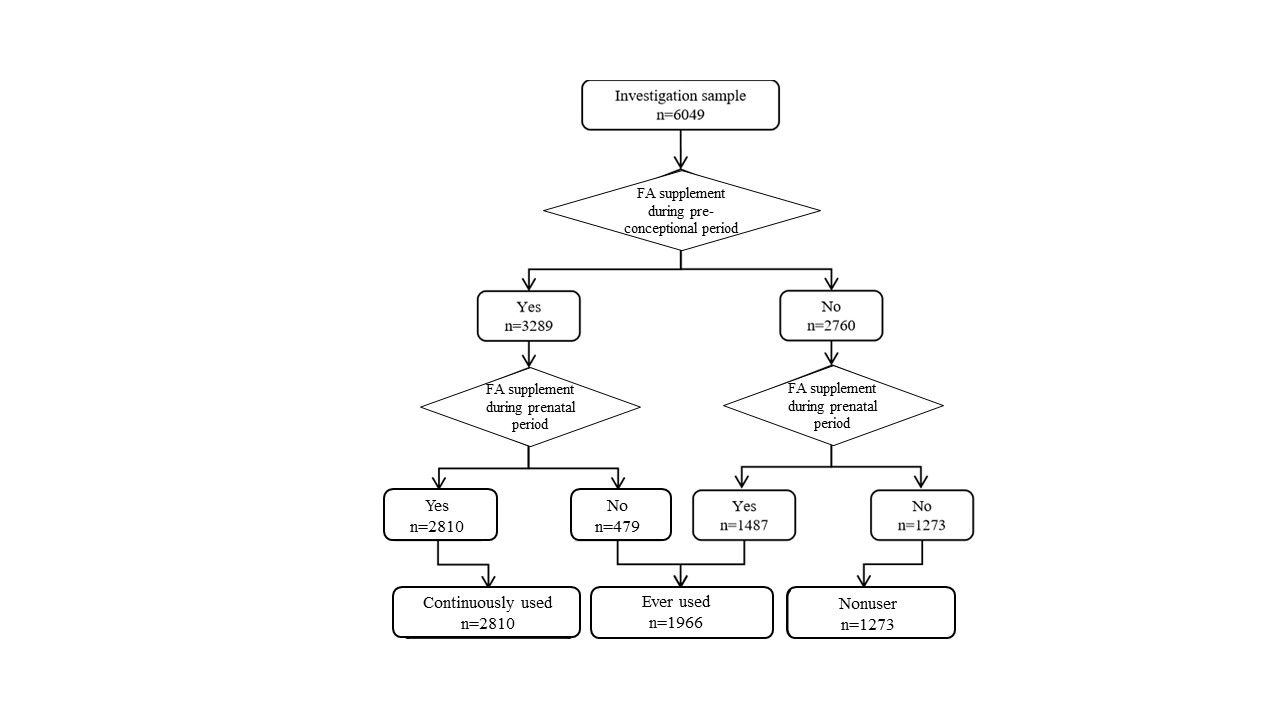


**Abbreviations**: FA folic acid.

**Supplementary Figure 2.** The Flowchart of 3 groups categorized with folic acid supplement during pre-conceptional and prenatal period

**Supplementary Table 1.** Summaries of previous observational studies regarding the associations of folic acid supplements with ASD

| Author | Study Period | Sample Size | Location | Outcomes | Exposure | | | OR/RR (95%CI) |
| --- | --- | --- | --- | --- | --- | --- | --- | --- |
|  |  |  |  |  | Measurements | Period of Intake | Types |  |
| **Case- control study** | | | | | | | | |
| Schmidt RJ,et al(1). | 2003-2009.7 | 429 ASD 278 TD 130 DD | USA | Diagnosed by Autism Diagnostic Interview–Revised (ADI-R) and the Autism Diagnostic Observation Schedule–Generic (ADOS) | Questionnaires (including the intake of folic acid and food information) | Beginning 3 mo before and throughout each month of pregnancy | A mean daily folic acid intake of <600 μg | Ref |
|  |  |  |  |  |  |  | ≥600 μg | 0.62 (0.42, 0.92) |
| Tan, et al(2) | 2019 | 416 ASD 201 TD | China | Diagnostic and Statistical Manual of Mental Disorders (DSM-5) criteria | Questionnaires | FA supplement during pregnancy [FA supplementation lasted 24 weeks, beginning 12 weeks before the last menstrual period (LMP) through 12 weeks after the LMP]. | User (the women who take on pill which contained 400μg FA daily) | Ref |
|  |  |  |  |  |  |  | Non-user | **1.905 (1.238–2.933)** |
| DeSoto & Hitlan(3) | 1994.1.1-1999.12.31(Center for disease control dataset) | 256 ASD 752 TD | USA | Administrative database | Questionnaires | During pregnancy | Non-user | Ref |
|  |  |  |  |  |  |  | FA supplementation/prenatal vitamin | **2.34 (1.14–4.82)** |
| Li, et al.(4) | Autism Clinical and Environmental Database (ACED). | 374 ASD 354 TD | China | Diagnostic and Statistical Manual of Mental Disorders. Fourth Edition, Text Revision (DSM-IV-TR) | Questionnaires |  | No FA supplementation | Ref |
|  |  |  |  |  |  | During pregnancy preparation | FA supplementation | 0.954(0.608-1.496) |
|  |  |  |  |  |  | During pregnancy | FA supplementation | 0.683(0.406-1.001) |
|  |  |  |  |  |  | During lactation | FA supplementation | 0.679(0.241-1.915) |
| **Cohort study** | | | | | | | | |
| Schmidt RJ, et al(5). | December 1, 2006, and June 30, 2015 | n = 332 children and their mother (n=305),  55 (22.8%) ASD, 60 non-TD 126 TD | USA | ADOS met the DSM-5 criteria for ASD Cognitive function was measured with the Mullen Scales of Early Learning (MSEL) | Self-reported | Maternal Prenatal vitamin use in First Month of Pregnancy | No use | Ref |
|  |  |  |  |  |  |  | FA supplement | **0.50 (0.30-0.81)** |
|  |  |  |  |  |  |  | Below recommendation (<600 μg) | Ref |
|  |  |  |  |  |  |  | At/above recommendation (≥600 μg) | **0.47 (0.29-0.75)** |
|  |  |  |  |  |  |  | Tertile 1 (0-57 μg) | Ref |
|  |  |  |  |  |  |  | Tertile 2 (80-800 μg) | **0.63 (0.40-0.98)** |
|  |  |  |  |  |  |  | Tertile 3 (805-4800 μg) | **0.42 (0.17-0.99)** |
| Virk, J.et al(6) | 1996–2002 | n=35 059 women, 552 (1.6%) ASD (8.1–11.4 years) | USA | ICD-10 | Self-reported | 4 weeks before to 8 weeks after the start of pregnancy | Supplement use (containing at least 400µg of FA) in at least 2 of the 4 weeks | Risk ratio: 1.06 (0.82–1.36) |
| Surén, P., et al.(7) | 2002.8-2012.3.31,Norwegian Mother and Child Cohort Study (MoBa) | n= 85 176 children, 270 (0.32%) ASD | Norway | Diagnoses are based on DSM-IV criteria using ADI-R and ADOS Autism Birth Cohort study,The registry contains ICD-10 codes determined by Norwegian specialist health services | Self-report | 4 weeks before to 8 weeks after the start of pregnancy | No FA supplementation | Ref |
|  |  |  |  |  |  |  | Multivitamin supplements (400µg of FA) /fish oil and FA | **0.61 (0.41–0.90)** |
| Raghavan, R., et al.(8) | Birth from 1998 to 2013 and followed up prospectively from 2003 to 2015 | 1257 mother-infant pairs, 86 (6.8%) ASD | USA | Electronic Medical Records (EMR) using ICD-9 codes | self-reported | Preconception | No use of multivitamins | Ref |
|  |  |  |  |  |  |  | Multivitamins supplements | 0.5 (0.1-2.1) |
|  |  |  |  |  |  | First Trimester | 3-5 times/week | Ref |
|  |  |  |  |  |  |  | 2times/week | **3.4 (1.6-7.2)** |
|  |  |  |  |  |  |  | >5 times/week | **2.3 (1.2-3.9)** |
|  |  |  |  |  |  | Second Trimster | 3-5 times/week | Ref |
|  |  |  |  |  |  |  | 2times/week | **3.8 (1.8-8.0)** |
|  |  |  |  |  |  |  | >5 times/week | **2.1 (1.2-3.6)** |
|  |  |  |  |  |  | Third Trimster | 3-5 times/week | Ref |
|  |  |  |  |  |  |  | 2times/week | **3.5 (1.7-7.4)** |
|  |  |  |  |  |  |  | >5 times/week | **2.1 (1.2-3.6)** |
| Levine, SZ., et al.(9) | A case-control cohort study of 45300 Israeli children born between January 1, 2003, and December 31, 2007, were followed up from birth to January 26, 2015, for the risk of ASD | 45300 (born to 26702 mothers), 527 (1.3%) ASD | Israel | The final ASD diagnosis is determined by a board-recognized developmental behavioral pediatrician. ICD-9 | Meuhedet Prescription Register | Before pregnancy but not during pregnancy | No FA and /or multivitamin supplementation | Ref |
|  |  |  |  |  |  |  | FA and/or multivitamin supplementation | **0.39 (0.30-0.50)** |
|  |  |  |  |  |  |  | FA supplementation | **0.56 (0.42-0.74)** |
|  |  |  |  |  |  | During but not before pregnancy | No FA and /or multivitamin supplementation | Ref |
|  |  |  |  |  |  |  | FA and/or multivitamin supplementation | **0.27 (0.22-0.33)** |
|  |  |  |  |  |  |  | FA supplementation | **0.32 (0.26-0.41)** |
| DeVilbiss, et al.(10) | Follow-up on 31 December 2011 and were born between 1996 and 2007. | n= 273 107 mother-child pairs, 158 (0.26%) ASD with intellectual disability | Sweden | ICD-10,DSM-IV | Anatomical Therapeutic Chemical (ATC) classification codes | First trimester | No use of multivitamins, irons or folic acid | Ref |
|  |  |  |  |  |  |  | Multivitamin supplements FA supplements(400µg) | **0.69(0.57-0.84); Sibling** control:0.77(0.52-1.15); Propensity score matched:0.68 (0.54-0.86) |
|  |  |  |  |  |  |  | Folic acid supplementation only | 1.20(0.71-2.01); Sibling control:0.94 (0.29-3.04); Propensity score matched:1.14 (0.64-2.04) |
| Steenweg-de Graaff, J., et al.(11) | NA | n = 5591 mothers of single live-born neonates, 3893 (70%) ASD | Netherlands | Parent-reported autistic traits using the Social Responsiveness Scale (SRS) short form; the Pervasive Developmental Problems (PDP) subscale of the Child Behaviour Checklist | Maternal plasma folate concentrations at 13 weeks of gestation and prenatal folic acid supplement use | Preconceptional within the first 10 weeks of pregnancy After the first 10 weeks of pregnancy | No FA supplementation | Ref |
|  |  |  |  |  |  |  | Preconceptional start | **B=-0.042(-0.068~-0.017)** |
|  |  |  |  |  |  |  | Start within the first 10 weeks of pregnancy | **B=-0.041(-0.066~-0.016)** |
|  |  |  |  |  |  |  | Start after the first 10 weeks of pregnancy | **B=-0.057(-0.089~-0.025)** |
| Nilsen RM, et al.(12) | The children were born in 1999–2007 | Medical Birth Registry of Norway: n = 507 856, 2072 (0.41%) ASD； | Norway | Administrative database based on ICD-10 | Self-reported | Prenatal FA supplements | No use | Ref |
|  |  |  |  |  |  |  | FA use | **0.86(0.78,0.95)** |
|  |  | Autism Birth Cohort: n = 89 836, 234 (0.26%) ASD |  |  |  |  | No use | Ref |
|  |  |  |  |  |  |  | FA use | **0.85(0.65,1.11)** |
| Abbreviations: ASD, autism spectrum disorder; SHS, secondhand smoke; OR, odds ratios; DSM-IV, The Diagnostic and Statistical Manual of Mental Disorders, Fourth Edition; DSM-5, The Diagnostic and Statistical Manual of Mental Disorders, Fifth Edition; DBH, The Dampness in Buildings and Health; ICD-10, International Classification of Diseases 10th Revision; | | | | | | | | |

**Supplementary Table 2** Demographic characteristics of 7928 study participants with or without the information of questionnaires

| Characteristics* | Uncomplete the questionnaires  (n=1879) | Complete the questionnaires  (n=6049) | Overall  (n=7928) | *P* value |
| --- | --- | --- | --- | --- |
|  | N (%) / Mean (SD) | N (%) / Mean (SD) | N (%) / Mean (SD) |  |
| Child age (in months) | 22.7 (3.8) | 22.7 (4.1) | 22.7 (4.0) | 0.71 |
| Child gender |  |  |  |  |
| Boy | 1027 (55.7%) | 3364 (55.6%) | 4391 (55.6%) | 0.97 |
| Girl | 818 (44.3%) | 2685 (44.4%) | 3503 (44.4%) |  |
| Only child |  |  |  |  |
| Yes | 514 (29.1%) | 1772 (29.3%) | 2286 (29.2%) | 0.87 |
| No | 1253 (70.9%) | 4277 (70.7%) | 5530 (70.8%) |  |
| Maternal education level* |  |  |  | 0.07 |
| Primary school and below | 441 (24.1%) | 1045 (17.3%) | 1486 (18.9%) |  |
| Middle school | 962 (52.5%) | 3188 (52.7%) | 4150 (52.7%) |  |
| College degree | 315 (17.2%) | 1407 (23.3%) | 1722 (21.8%) |  |
| Advanced degree | 115 (6.3%) | 409 (6.8%) | 524 (6.6%) |  |

**Abbreviations**: SD, Standard deviation

*Among those refused to participate, 34 children with unknown gender, 112 without only child information, 148 with unknown maternal education level.

**Supplementary Table 3** Prevalence of ASD associated with maternal folic acid supplement among toddlers aged16-30 Months

| Timing of folic acid supplement |  | No. |  | Children with ASD | |
| --- | --- | --- | --- | --- | --- |
|  |  |  |  |  |  |
|  |  |  |  | n (%) |  |
| **Total** |  | 6049 |  | 71(1.2) |  |
| **Pre-conceptional period** |  |  |  |  |  |
| Yes |  | 3289 |  | 34(1.0) |  |
| No |  | 2760 |  | 37(1.3) |  |
| **Prenatal period** |  |  |  |  |  |
| Yes |  | 4297 |  | 47(1.1) |  |
| No |  | 1752 |  | 24(1.4) |  |
| **Pre-conceptional and prenatal period** |  |  |  |  |  |
| Continuously used |  | 2810 |  | 29(1.0) |  |
| Ever used |  | 1966 |  | 23(1.2) |  |
| Nonuser |  | 1273 |  | 19(1.5) |  |

**Abbreviations**: ASD, autism spectrum disorder

**Supplementary Table 4** The Firth’s Bias-Reduced Logistic Regression of the associations regarding pre-conceptional and prenatal folic acid supplement with ASD among toddlers aged 16-30 months

| Timing of folic acid supplement |  | Adjusted model ^a^ | |
| --- | --- | --- | --- |
|  |  | OR (95%CI) | *P* value |
| **Pre-conceptional period** | |  |  |
| Yes |  | 1[Reference] |  |
| No |  | 1.47(0.90, 2.38) | 0.12 |
| **Prenatal period** | |  |  |
| Yes |  | 1[Reference] |  |
| No |  | **2.53(1.42, 4.39)** | **< 0.001** |
| **Pre-conceptional and prenatal period** | |  |  |
| Continuously used |  | 1[Reference] |  |
| Ever used |  | 1.23(0.70, 2.15) | 0.47 |
| Nonuser |  | **2.90(1.49, 5.55)** | **<0.001** |

^a^ Adjusted model: adjusted for child age, gender, only child, maternal age, maternal education level, household income, research area, ethnic background, preterm birth, overweight/obesity before pregnancy, pregnant complications, depressive symptoms during pregnancy, second-hand smoke during pregnancy.

**Supplementary Table 5** Sensitivity analyses of the associations regarding pre-conceptional and prenatal folic acid supplement with ASD among toddlers aged 16-30 months restricting in boys

| Timing of folic acid supplement |  | Adjusted model^b^ | |
| --- | --- | --- | --- |
|  |  | OR (95%CI) | *P* value |
| **Pre-conceptional period** | |  |  |
| Yes |  | 1[Reference] |  |
| No |  | 1.53(0.90, 2.61) | 0.11 |
| **Prenatal period** | |  |  |
| Yes |  | 1[Reference] |  |
| No |  | **2.39(1.27, 4.38)** | **0.01** |
| **Pre-conceptional and prenatal period** | |  |  |
| Continuously used |  | 1[Reference] |  |
| Ever used |  | 1.32(0.71, 2.41) | 0.37 |
| Nonuser |  | **2.82(1.35, 5.75)** | **< 0.001** |

^b^ Adjusted model: adjusted for child age, only child, maternal age, maternal education level, household income, research area, ethnic background, preterm birth, overweight/obesity before pregnancy, pregnant complications, depressive symptoms during pregnancy, second-hand smoke during pregnancy.

**Supplementary Table 6** Sensitivity analyses of the associations regarding pre-conceptional and prenatal folic acid supplement with ASD among toddlers aged 16-30 months restricting in full-term infants

| Timing of folic acid supplement |  | Adjusted model^c^ | |
| --- | --- | --- | --- |
|  |  | OR (95%CI) | *P* value |
| **Pre-conceptional period** | |  |  |
| Yes |  | 1[Reference] |  |
| No |  | 1.38(0.83, 2.28) | 0.21 |
| **Prenatal period** | |  |  |
| Yes |  | 1[Reference] |  |
| No |  | **2.71(1.49, 4.81)** | **< 0.001** |
| **Pre-conceptional and prenatal period** | |  |  |
| Continuously used |  | 1[Reference] |  |
| Ever used |  | 1.16(0.64, 2.07) | 0.63 |
| Nonuser |  | **2.91(1.45, 5.71)** | **< 0.001** |

^c^ Adjusted model: adjusted for child age, child gender, only child, maternal age, maternal education level, household income, research area, ethnic background, complications during pregnancy, depression during pregnancy, second-hand smoke during pregnancy, overweight or obesity before pregnancy.

**Supplementary Table 7** Sensitivity analyses of the associations regarding pre-conceptional and prenatal folic acid supplement with ASD among toddlers aged 16-30 months with mothers of no complications during pregnancy

| Timing of folic acid supplement |  | Adjusted model^d^ | |
| --- | --- | --- | --- |
|  |  | OR (95%CI) | *P* value |
| **Pre-conceptional period** | |  |  |
| Yes |  | 1[Reference] |  |
| No |  | 1.40(0.83, 2.39) | 0.21 |
| **Prenatal period** | |  |  |
| Yes |  | 1[Reference] |  |
| No |  | **2.98(1.61, 5.35)** | **< 0.001** |
| **Pre-conceptional and prenatal period** | |  |  |
| Continuously used |  | 1[Reference] |  |
| Ever used |  | 1.17(0.62, 2.17) | 0.62 |
| Nonuser |  | **3.18(1.55, 6.38)** | **< 0.001** |

^d^ Adjusted model: adjusted for child age, child gender, only child, maternal age, maternal education level, household income, research area, ethnic background, preterm birth, overweight/obesity before pregnancy, depressive symptoms during pregnancy, second-hand smoke during pregnancy.

**Supplementary Table 8** Sensitivity analyses of the associations regarding pre-conceptional and prenatal folic acid supplement with ASD among toddlers aged 16-30 months with mothers of no Pre-pregnancy overweight/obesity

| Timing of folic acid supplement |  | Adjusted model^e^ | |
| --- | --- | --- | --- |
|  |  | OR (95%CI) | *P* value |
| **Pre-conceptional period** | |  |  |
| Yes |  | 1[Reference] |  |
| No |  | 1.38(0.83, 2.32) | 0.22 |
| **Prenatal period** | |  |  |
| Yes |  | 1[Reference] |  |
| No |  | **2.39(1.28, 4.32)** | **< 0.001** |
| **Pre-conceptional and prenatal period** | |  |  |
| Continuously used |  | 1[Reference] |  |
| Ever used |  | 1.24(0.68, 2.23) | 0.47 |
| Nonuser |  | **2.64(1.27, 5.34)** | **0.01** |

^e^ Adjusted model: adjusted for child age, child gender, only child, maternal age, maternal education level, household income, research area, ethnic background, preterm birth, pregnant complications, depressive symptoms during pregnancy, second-hand smoke during pregnancy.

**Reference**

1. Schmidt RJ, Tancredi DJ, Ozonoff S, Hansen RL, Hartiala J, Allayee H, et al. Maternal periconceptional folic acid intake and risk of autism spectrum disorders and developmental delay in the CHARGE (CHildhood Autism Risks from Genetics and Environment) case-control study. Am J Clin Nutr. 2012;96(1):80-9.

2. Tan M, Yang T, Zhu J, Li Q, Lai X, Li Y, et al. Maternal folic acid and micronutrient supplementation is associated with vitamin levels and symptoms in children with autism spectrum disorders. Reprod Toxicol. 2020;91:109-15.

3. Hitlan MCDaRT. Synthetic folic acid supplementation during pregnancy may increase the risk of developing autism. Journal of Pediatric Biochemistry. 2012;2:251-61.

4. Li YM, Shen YD, Li YJ, Xun GL, Liu H, Wu RR, et al. Maternal dietary patterns, supplements intake and autism spectrum disorders: A preliminary case-control study. Medicine (Baltimore). 2018;97(52):e13902.

5. Schmidt RJ, Iosif AM, Guerrero Angel E, Ozonoff S. Association of Maternal Prenatal Vitamin Use With Risk for Autism Spectrum Disorder Recurrence in Young Siblings. JAMA Psychiatry. 2019;76(4):391-8.

6. Virk J, Liew Z, Olsen J, Nohr EA, Catov JM, Ritz B. Preconceptional and prenatal supplementary folic acid and multivitamin intake and autism spectrum disorders. Autism. 2016;20(6):710-8.

7. Suren P, Roth C, Bresnahan M, Haugen M, Hornig M, Hirtz D, et al. Association between maternal use of folic acid supplements and risk of autism spectrum disorders in children. JAMA. 2013;309(6):570-7.

8. Raghavan R, Riley AW, Volk H, Caruso D, Hironaka L, Sices L, et al. Maternal Multivitamin Intake, Plasma Folate and Vitamin B12 Levels and Autism Spectrum Disorder Risk in Offspring. Paediatr Perinat Epidemiol. 2018;32(1):100-11.

9. Levine SZ, Kodesh A, Viktorin A, Smith L, Uher R, Reichenberg A, et al. Association of Maternal Use of Folic Acid and Multivitamin Supplements in the Periods Before and During Pregnancy With the Risk of Autism Spectrum Disorder in Offspring. JAMA Psychiatry. 2018;75(2):176-84.

10. DeVilbiss EA, Magnusson C, Gardner RM, Rai D, Newschaffer CJ, Lyall K, et al. Antenatal nutritional supplementation and autism spectrum disorders in the Stockholm youth cohort: population based cohort study. BMJ. 2017;359:j4273.

11. Steenweg-de Graaff J, Ghassabian A, Jaddoe VW, Tiemeier H, Roza SJ. Folate concentrations during pregnancy and autistic traits in the offspring. The Generation R Study. Eur J Public Health. 2015;25(3):431-3.

12. Nilsen RM, Suren P, Gunnes N, Alsaker ER, Bresnahan M, Hirtz D, et al. Analysis of self-selection bias in a population-based cohort study of autism spectrum disorders. Paediatr Perinat Epidemiol. 2013;27(6):553-63.
